# Congenital Anomaly of the Peroneus Brevis Tendon With a Bifid Digastric Variant Lacking Insertion on the Fifth Metatarsal Base: A Case Report

**DOI:** 10.1177/24730114261485215

**Published:** 2026-09-09

**Authors:** Sayi P. Boddu, Alex C. Chang, Brady K. Huang, Ian M. Foran

**Affiliations:** 1 Department of Orthopaedic Surgery, University of California, San Diego, CA, USA; 2 Department of Radiology, University of California, San Diego, CA, USA

**Keywords:** peroneus, brevis, peroneal, variant, tendon disorders, ankle stability, congenital, fibularis, digastric, bifid

## Introduction

Multiple peroneal tendon variants have been described in the medical literature, including the peroneus quartus and peroneus digiti minimi accessory tendons.^1-3^ Recognition of tendon anomalies is essential to avoid misdiagnosis and improper treatment. To our knowledge, no variant has been described in which the peroneus brevis does not insert on the fifth metatarsal base. Here, we present a case with a bifid peroneus brevis tendon where one slip inserted on the calcaneus and the other slip had a second muscle belly that appeared to blend with the extensor digitorum brevis muscle.

## Case Report

A 31-year-old man with no medical history presented for evaluation of an acute left Achilles tendon rupture following a noncontact rugby injury. Incidentally, he also endorsed a longstanding history of recurrent left inversion-type ankle sprains. There was no history of prior ankle fracture. He noted similar though milder instability of the contralateral right ankle.

Physical examination indicated Achilles tendon rupture. Eversion strength was 5/5. There was no tenderness along the peroneal tendons. Anterior drawer, anterolateral drawer, and talar tilt testing revealed increased laxity compared with the contralateral side.

**Figure 1.**
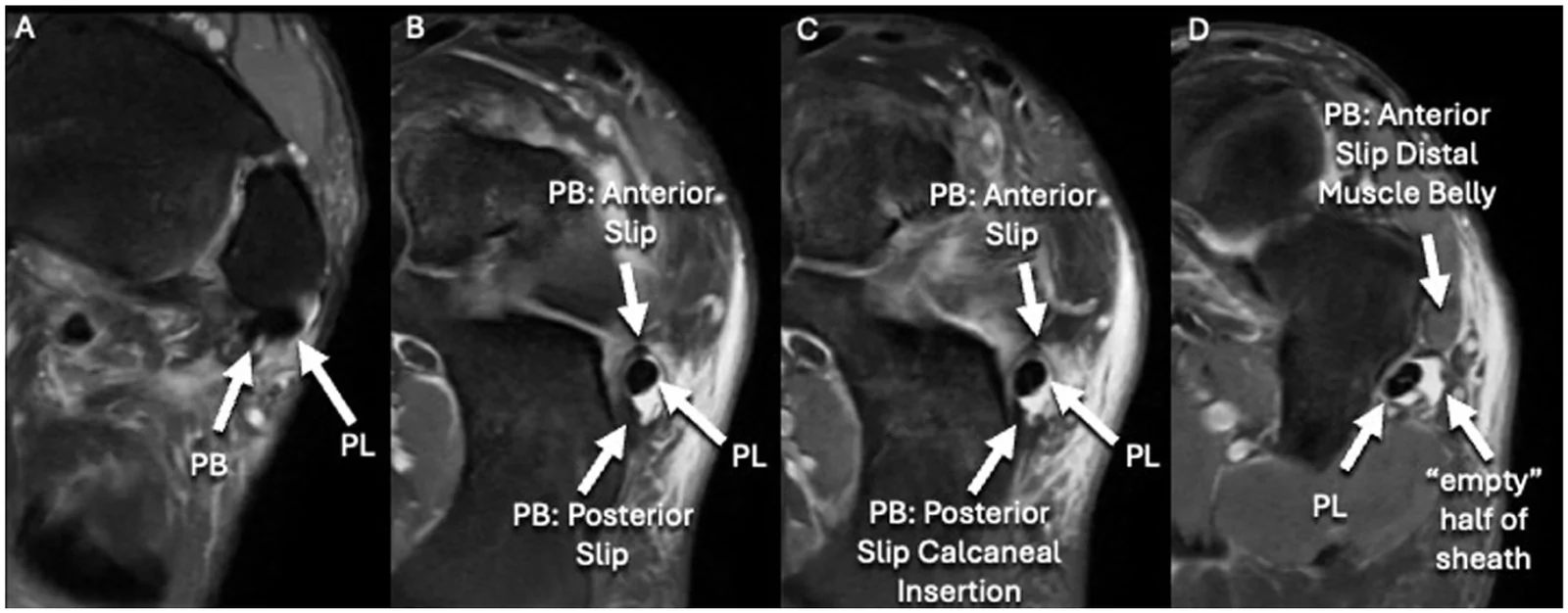
Axial oblique proton density fat-suppressed MRI images of the left ankle (A-D, proximal to distal). The diminutive peroneus brevis bifurcates proximal to the fibular tip into a posterior slip (inserting on the lateral calcaneus) and an anterior slip with a distinct distal muscle belly forming a digastric configuration. The peroneal tendon sheath is half-empty distally adjacent to the peroneus longus, where a normal-caliber peroneus brevis would be expected. PB, peroneus brevis; PL, peroneus longus.

**Figure 2.**
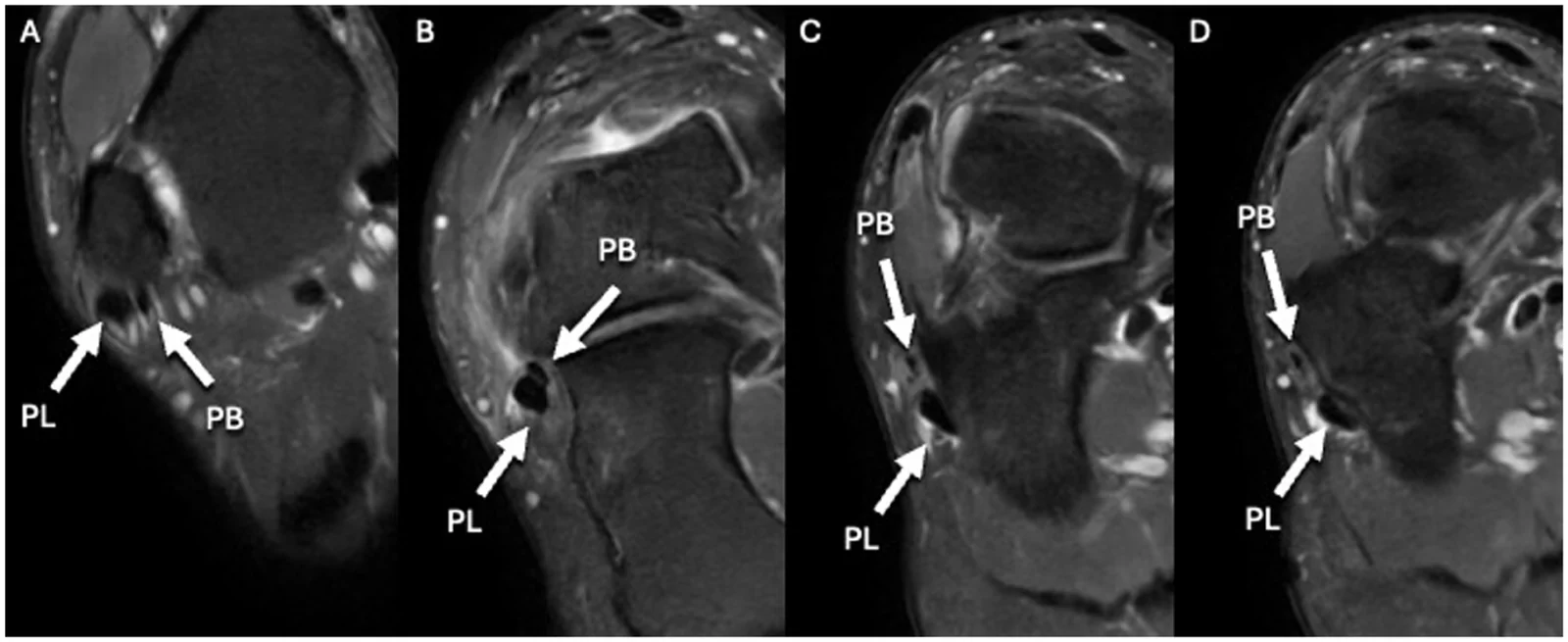
Axial oblique proton density fat-suppressed MRI images of the right ankle (A–D, proximal to distal). The peroneus brevis tendon courses “normally” without a bifid configuration but is diminutive distally, becoming difficult to trace near the calcaneocuboid joint. PB, peroneus brevis; PL, peroneus longus.

Magnetic resonance imaging (MRI) of the left ankle confirmed a complete midsubstance Achilles tendon rupture. The initial radiology report described a chronic-appearing complete avulsion of the peroneus brevis tendon from its fifth metatarsal insertion with retraction to the fibular tip, along with high-grade chronic tearing of the anterior talofibular ligament (Figure 1). Given the rarity of a chronic peroneus brevis rupture in a young patient without prior injury other than recurrent ankle sprains, a contralateral right ankle MRI was obtained. The right ankle MRI also demonstrated a diminutive appearance of the peroneus brevis tendon, possibly reflecting partial tearing (Figure 2). The bilateral nature of peroneal tendon abnormalities raised suspicion of a congenital tendon anomaly.

**Figure 3.**
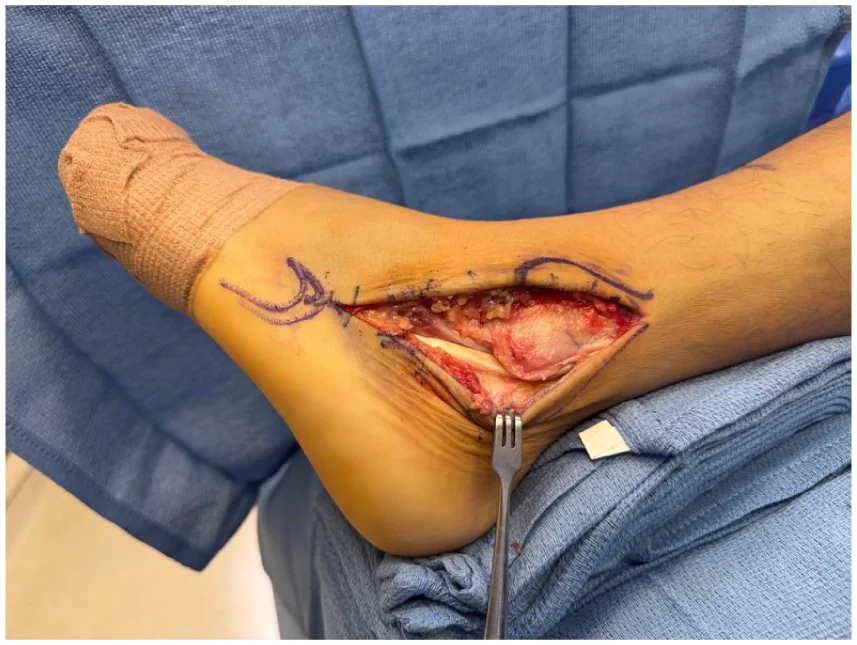
Peroneus longus tendon visualized with apparent hypertrophy.

The patient elected to proceed with operative management to address the Achilles tendon, the chronic lateral ligament instability, and any potential peroneal tendon pathology. Surgery was performed under general anesthesia with the patient initially in the supine position. A curvilinear longitudinal incision was made 3 cm proximal to the fibular tip to 3 cm proximal to the fifth metatarsal base. After dissection, the peroneus longus was identified and appeared hypertrophied without discrete pathology. (Figure 3).

**Figure 4.**
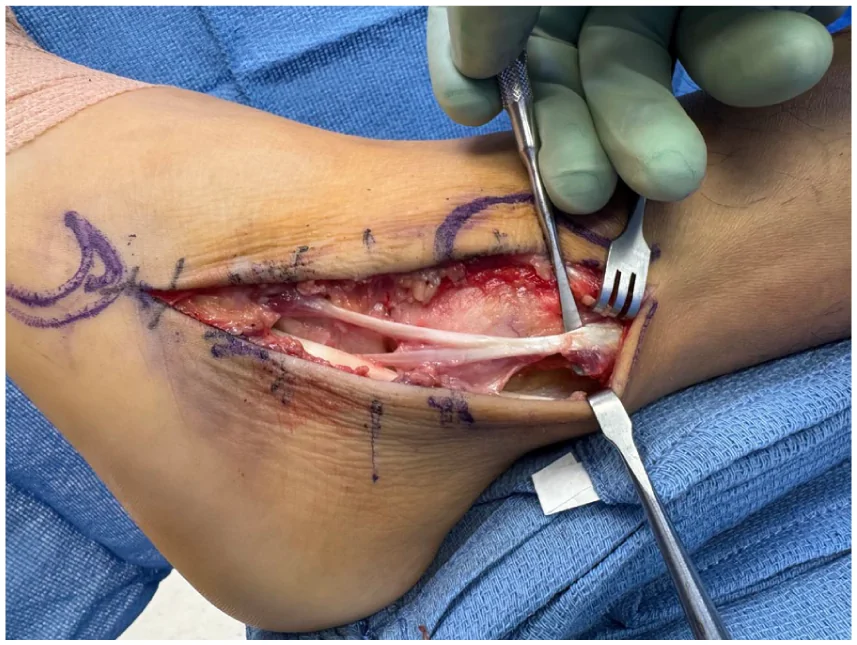
Accessory tendon with the proximal muscle belly.

**Figure 5.**
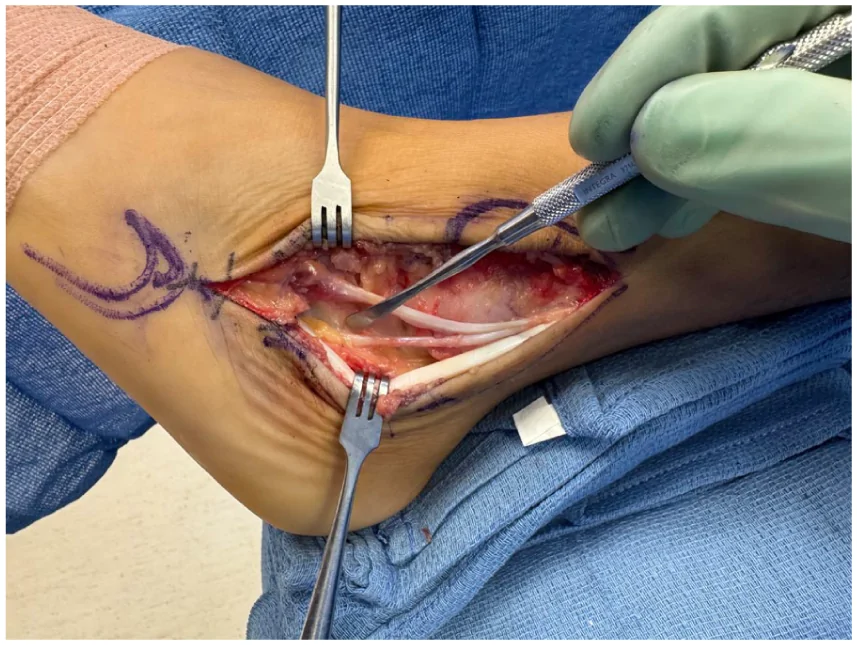
Posterior slip of accessory tendon appearing to insert on calcaneus.

**Figure 6.**
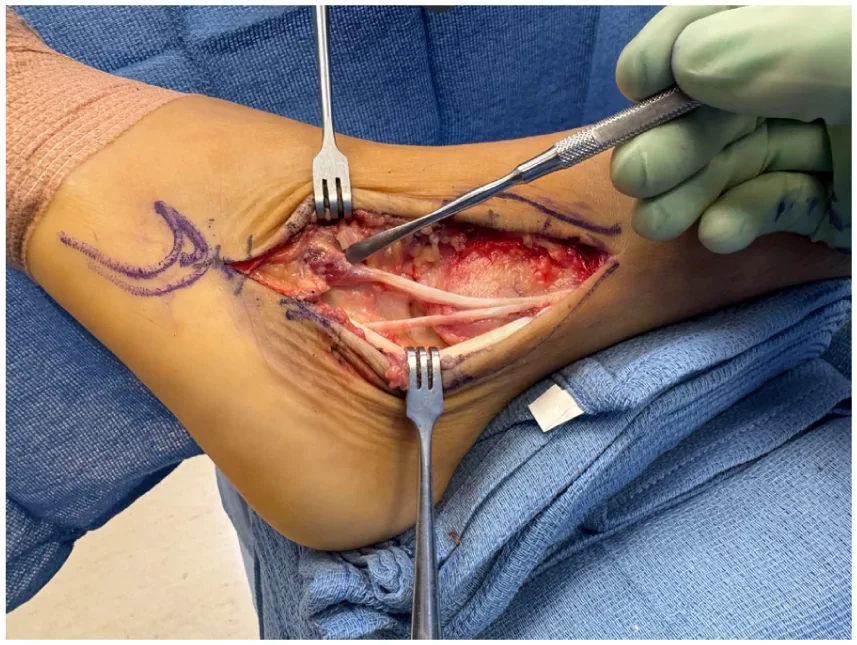
Anterior slip of accessory tendon with distal muscle belly coursing anteriorly.

The peroneus brevis tendon proximal to the lateral malleolus was diminutive, and just distal to its muscle belly, the tendon bifurcated 1 cm proximal to the fibular tip (Figure 4). There was no evidence of traumatic injury, tearing, or fraying of these tendon slips. One slip inserted onto the lateral calcaneus in a location similar to a peroneus quartus tendon (Figure 5). The second slip coursed anteriorly and possessed a distinct, separate distal muscle belly, forming a digastric configuration (Figure 6). This appeared to blend with the extensor digitorum muscle. On retrospective analysis of the preoperative MRI, these distinct slips and the digastric muscle belly are identified (Figure 1). Neither of these tendons were directed toward the normal insertion of the peroneus brevis tendon at the fifth metatarsal base. Given the lack of apparent injury to these tendon slips, they were recognized as an anatomic variant, and no reconstruction was performed to prevent any iatrogenic injury to the patient.

Attention then turned to lateral ligament repair via a modified Brostrom procedure. The patient was then repositioned prone for a minimally invasive Achilles tendon repair. The patient was discharged without complication.

At 2.5 months of follow-up, the patient was ambulating without assistive devices, had no pain, and had no feelings of instability. On examination, his anterior drawer and talar tilt testing was normal.

## Discussion

To our knowledge, this is the first reported case of a congenital peroneus brevis anatomic anomaly with a novel bifid digastric variant with an absent fifth metatarsal base insertion. In this case, the MRI was difficult to interpret because of the novelty of this variant. It should be noted that MRI also can have limited sensitivity and specificity for peroneal tendon pathology.^4^ Review of the MRI images postoperatively revealed a partially empty peroneal tendon sheath along the expected path of a “normal” peroneal brevis tendon (Figure 1). This finding was not symmetric on the contralateral ankle, which demonstrated an abnormally diminutive tendon but along the standard course (Figure 2). Bilateral, although asymmetric, peroneus brevis tendon pathology on MRI without any history of trauma in a young patient strongly supports a congenital variant. Hypertrophy of the peroneus longus, as observed here, may represent a compensatory adaptation.

There is one prior report of peroneus brevis agenesis documented by Hodo et al^5^ in a patient with a teratogenic syndrome, where MRI revealed absence of both the posterior tibialis and peroneus brevis. Accessory muscles such as the peroneus quartus and peroneus digiti quinti are well described. However, both variants occur alongside an existing peroneus brevis tendon inserting on the fifth metatarsal. Multiple large MRI series catalog these peroneal variants without encountering absence of the peroneus brevis.^6,7^ Similarly, Olewnik et al’s^8^ morphologic classification delineates peroneus brevis morphology but does not accommodate the presence of a diminutive peroneus brevis without a fifth metatarsal insertion.

Digastric muscle bellies are rare. One such muscle known for and named after this configuration is the digastric muscle found in the suprahyoid region of the neck. Although there are no known standard anatomical structures with this configuration in the lower extremity, Olewnik et al^8^ describe a rare bifid peroneus brevis tendon variant (IIC) in which one tendon slip attached to the fifth metatarsal base and the other slip attached to the peroneus tertius tendon and gave rise to a distal muscle belly interpreted as the “fourth dorsal interosseous muscle.”

## Conclusion

The MRI appearance of an absent peroneus brevis tendon can be indistinguishable from chronic traumatic rupture. Intraoperative recognition of the anomaly prevented unnecessary distal wound extension and a futile reconstruction attempt. This case expands the known spectrum of peroneal anatomic variation, falls outside current classification frameworks, and underscores the importance of critical evaluation of preoperative imaging in atypical presentations.

## Supplemental Material

sj-pdf-1-fao-10.1177_24730114261485215 – Supplemental material for Congenital Anomaly of the Peroneus Brevis Tendon With a Bifid Digastric Variant Lacking Insertion on the Fifth Metatarsal Base: A Case Report Supplemental material, sj-pdf-1-fao-10.1177_24730114261485215 for Congenital Anomaly of the Peroneus Brevis Tendon With a Bifid Digastric Variant Lacking Insertion on the Fifth Metatarsal Base: A Case Report by Sayi P. Boddu, Alex C. Chang, Brady K. Huang and Ian M. Foran in Foot & Ankle Orthopaedics
