# Supplementary material for: Congenital Anomaly of the Peroneus Brevis Tendon With a Bifid Digastric Variant Lacking Insertion on the Fifth Metatarsal Base: A Case Report
Source: Foot Ankle Orthop. 2026 Sep 9;11(3):24730114261485215. doi: 10.1177/24730114261485215 (PMC13558994; doi:10.1177/24730114261485215)
Supplement: Supplementary file 1 [file sj-pdf-1-fao-10.1177_24730114261485215.pdf]

## Conflict of Interest (COI) Disclosure Form FAI / FAO

**Purpose:**
All authors submitting a manuscript to this peer-reviewed journal **must disclose any conflicts of interest (COI) RELATED DIRECTLY to the submitted paper**. **No other disclosures are required or desired.**

A conflict of interest typically involves **financial relationships (most commonly payments to surgeons or authors)** that could reasonably be perceived as influencing the submitted work, whether **received in the past or anticipated in the future**.

---

| | |
|---|---|
| **Author Name:** | **Sayi Boddu** |
| **Manuscript Title:** | **Congenital Anomaly of the Peroneus Brevis Tendon with a Bifid Digastric Variant Lacking Insertion on the Fifth Metatarsal Base: A Case Report** |
| **Date:** | **7/12/26** |

**Conflict of Interest Disclosure:**

**Do you or an immediate family member (spouse, domestic partner, or dependent child) have financial conflicts of interest valued over $500 related to this manuscript or its subject matter? (Do NOT list any unrelated disclosures.)**
(Examples include consulting fees, honoraria, royalties, stock or stock options, research funding, paid advisory roles, product or material support, or intellectual property interests (e.g., potential future royalties) valued over $500, received within the past three years or expected in the future.)

☒ **No — I have no conflicts of interest to disclose.**

☐ **Yes — I have a conflict of interest to disclose.**
If **Yes**, please briefly describe the financial relationship(s) below, including the company/entity involved and the nature of the payment or support:

Click or tap here to enter text.

☒ **I, the undersigned author, personally completed this disclosure and attest to its accuracy. I understand that I am solely responsible for its content.**

**Updates:** If a relevant conflict of interest arises or changes after submission and before publication, an updated disclosure must be provided.

**Publication of Disclosure:** All disclosed conflicts of interest will be published with the article and completed forms will be made available as an online supplement.

## Conflict of Interest (COI) Disclosure Form FAI / FAO

**Purpose:**
All authors submitting a manuscript to this peer-reviewed journal **must disclose any conflicts of interest (COI) RELATED DIRECTLY to the submitted paper**. **No other disclosures are required or desired.**

A conflict of interest typically involves **financial relationships (most commonly payments to surgeons or authors)** that could reasonably be perceived as influencing the submitted work, whether **received in the past or anticipated in the future**.

---

| | |
|---|---|
| **Author Name:** | **Alex Chang** |
| **Manuscript Title:** | **Congenital Anomaly of the Peroneus Brevis Tendon with a Bifid Digastric Variant Lacking Insertion on the Fifth Metatarsal Base: A Case Report** |
| **Date:** | **7/13/26** |

**Conflict of Interest Disclosure:**

**Do you or an immediate family member (spouse, domestic partner, or dependent child) have financial conflicts of interest valued over $500 <u>related to this manuscript or its subject matter</u>? (Do NOT list any unrelated disclosures.)**
(Examples include consulting fees, honoraria, royalties, stock or stock options, research funding, paid advisory roles, product or material support, or intellectual property interests (e.g., potential future royalties) valued over $500, received within the past three years or expected in the future.)

☒ **No — I have no conflicts of interest to disclose.**

☐ **Yes — I have a conflict of interest to disclose.**
If **Yes**, please briefly describe the financial relationship(s) below, including the company/entity involved and the nature of the payment or support:

| |
|---|
| Click or tap here to enter text. |

☒ **I, the undersigned author, personally completed this disclosure and attest to its accuracy. I understand that I am solely responsible for its content.**

**Updates:** If a relevant conflict of interest arises or changes after submission and before publication, an updated disclosure must be provided.

**Publication of Disclosure:** All disclosed conflicts of interest will be published with the article and completed forms will be made available as an online supplement.

## Conflict of Interest (COI) Disclosure Form FAI / FAO

**Purpose:**
All authors submitting a manuscript to this peer-reviewed journal **must disclose any conflicts of interest (COI) RELATED DIRECTLY to the submitted paper**. **No other disclosures are required or desired.**

A conflict of interest typically involves **financial relationships (most commonly payments to surgeons or authors)** that could reasonably be perceived as influencing the submitted work, whether **received in the past or anticipated in the future**.

---

| | |
|---|---|
| **Author Name:** | **Brady Huang** |
| **Manuscript Title:** | **Congenital Anomaly of the Peroneus Brevis Tendon with a Bifid Digastric Variant Lacking Insertion on the Fifth Metatarsal Base: A Case Report** |
| **Date:** | **7/13/26** |

**Conflict of Interest Disclosure:**

**Do you or an immediate family member (spouse, domestic partner, or dependent child) have financial conflicts of interest valued over $500 <u>related to this manuscript or its subject matter</u>? (Do NOT list any unrelated disclosures.)**
(Examples include consulting fees, honoraria, royalties, stock or stock options, research funding, paid advisory roles, product or material support, or intellectual property interests (e.g., potential future royalties) valued over $500, received within the past three years or expected in the future.)

☒ **No — I have no conflicts of interest to disclose.**

☐ **Yes — I have a conflict of interest to disclose.**
If **Yes**, please briefly describe the financial relationship(s) below, including the company/entity involved and the nature of the payment or support:

Click or tap here to enter text.

☒ **I, the undersigned author, personally completed this disclosure and attest to its accuracy. I understand that I am solely responsible for its content.**

**Updates:** If a relevant conflict of interest arises or changes after submission and before publication, an updated disclosure must be provided.

**Publication of Disclosure:** All disclosed conflicts of interest will be published with the article and completed forms will be made available as an online supplement.

## Conflict of Interest (COI) Disclosure Form FAI / FAO

**Purpose:**
All authors submitting a manuscript to this peer-reviewed journal **must disclose any conflicts of interest (COI) RELATED DIRECTLY to the submitted paper**. **No other disclosures are required or desired.**

A conflict of interest typically involves **financial relationships (most commonly payments to surgeons or authors)** that could reasonably be perceived as influencing the submitted work, whether **received in the past or anticipated in the future**.

---

| | |
|---|---|
| **Author Name:** | **Ian Foran** |
| **Manuscript Title:** | **Congenital Anomaly of the Peroneus Brevis Tendon with a Bifid Digastric Variant Lacking Insertion on the Fifth Metatarsal Base: A Case Report** |
| **Date:** | **7/13/26** |

**Conflict of Interest Disclosure:**

**Do you or an immediate family member (spouse, domestic partner, or dependent child) have financial conflicts of interest valued over $500 <u>related to this manuscript or its subject matter</u>? (Do NOT list any unrelated disclosures.)**
(Examples include consulting fees, honoraria, royalties, stock or stock options, research funding, paid advisory roles, product or material support, or intellectual property interests (e.g., potential future royalties) valued over $500, received within the past three years or expected in the future.)

☒ **No — I have no conflicts of interest to disclose.**

☐ **Yes — I have a conflict of interest to disclose.**
If **Yes**, please briefly describe the financial relationship(s) below, including the company/entity involved and the nature of the payment or support:

| |
|---|
| Click or tap here to enter text. |

☒ **I, the undersigned author, personally completed this disclosure and attest to its accuracy. I understand that I am solely responsible for its content.**

**Updates:** If a relevant conflict of interest arises or changes after submission and before publication, an updated disclosure must be provided.

**Publication of Disclosure:** All disclosed conflicts of interest will be published with the article and completed forms will be made available as an online supplement.
